# Backwash Ileitis—From Pathogenesis to Clinical Significance: Literature Review

**DOI:** 10.3390/life15040567

**Published:** 2025-03-31

**Authors:** Alina-Ecaterina Jucan, Otilia Nedelciuc, Vasile-Claudiu Mihai, Mihaela Dranga, Mihaela-Cristiana Andronic, Simona-Stefania Juncu, Georgiana-Elena Sarbu, Ioana-Ruxandra Mihai, Andrei Andronic, Irina Ciortescu, Vasile Drug, Cristina Cijevschi Prelipcean, Catalina Mihai

**Affiliations:** 1 Department of Gastroenterology, “Grigore T. Popa” University of Medicine and Pharmacy, 700115 Iasi, Romania; ghiata.alina-ecaterina@d.umfiasi.ro (A.-E.J.); mihaela_dra@yahoo.com (M.D.); andronic_mihaela-cristiana@d.umfiasi.ro (M.-C.A.); juncu_simona-stefania@d.umfiasi.ro (S.-S.J.); georgiana_ciobanu23@yahoo.com (G.-E.S.); irinaciortescu@yahoo.com (I.C.); vasidrug@email.com (V.D.); catalina.mihai@umfiasi.ro (C.M.); 2 Department of Gastroenterology, Saint Spiridon County Hospital, 700111 Iasi, Romania; cristina.cijevschi.prelipcean@umfiasi.ro; 3 Department of Radiology, Faculty of Medicine, “Grigore T. Popa” University of Medicine and Pharmacy, 700115 Iasi, Romania; mihai_vasile-claudiu@d.umfiasi.ro; 4 Department of Rheumatology and Rehabilitation, Faculty of Medicine, “Grigore T. Popa” University of Medicine and Pharmacy, 700115 Iasi, Romania; ioana-ruxandra_mihai@umfiasi.ro; 5 Discipline of Medical Semiology, Medical Department I, “Grigore T. Popa” University of Medicine and Pharmacy, 700115 Iasi, Romania; andrei.andronic@umfiasi.ro

**Keywords:** backwash ileitis, ulcerative colitis, primary sclerosing cholangitis, proctocolectomy, colorectal cancer

## Abstract

Backwash ileitis (BWI) refers to inflammation in the distal ileum in patients with extensive ulcerative colitis (UC) that is thought to be caused by a “reflux” or “backwash” of colonic contents. In the absence of well-defined diagnostic criteria for BWI, more recently, the term UC-associated ileitis was proposed in favor of the backwash theory, which questions the existence of backwash ileitis as a distinct disease-specific subset of patients. Moreover, distinguishing UC-associated BWI from terminal ileitis of Crohn’s disease or other conditions could be a diagnostic challenge and significantly affect treatment management. Therefore, clinical, endoscopic, histologic, and imagistic diagnostic features may aid in making this distinction. This literature review related to BWI outlines the hypothesis that the ileum may also become involved in UC as a primary manifestation of UC based on recent findings. This study also highlights the possibility that associated ileitis in UC patients may represent a potential risk factor for neoplasia, a positive association with primary sclerosing cholangitis, and a higher risk for the subsequent development of pouchitis after restorative proctocolectomy. It synthesizes recent key findings and highlights areas for further research.

## 1. Introduction

Ulcerative colitis (UC) and Crohn’s disease (CD) are chronic relapsing and recurring inflammatory bowel diseases of the gastrointestinal tract. According to the European Crohn’s and Colitis Organization (ECCO) guidelines, the quality of the diagnosis of CD and UC was improved by the recent ECCO consensus position statements based on clinical, biochemical, endoscopic, and recent histological features [1,2].

UC is a disease that begins in the rectum and may extend proximally, in continuity, involving a variable colon length. Most patients with UC do not have an extension of the inflammatory process into the terminal ileum (TI), and the involvement of TI would generally raise suspicion for CD. The original interpretation of the term commonly known as backwash ileitis (BWI) was given by the extension of inflammation and injury to the terminal portion of the ileum in patients with extensive UC resulting from a reaction to reflux of colonic contents [3]. Due to recent evidence regarding this concept, the controversy over whether backwash ileitis is a misnomer or considered a primary manifestation of UC involving the ileum is still debatable [4].

Associated BWI in UC patients is not necessarily a benign phenomenon; it seems to influence the natural course of UC and appears to predispose patients to a more refractory course of the disease. Findings show a strong association between BWI and primary sclerosing cholangitis (PSC) in UC patients [5]. Recent findings also substantiate the link between BWI and an increased risk of colorectal cancer in proctocolectomy specimens. Moreover, an extensive UC BWI was reported as a risk factor for pouchitis [6,7].

Therefore, this literature review aims to systematically highlight the evidence regarding the contemporary and revised definition of BWI by distinguishing BWI from CD or other ileitis, including clinical, endoscopic, histological, and imaging data. The presence of BWI, PSC, and pouchitis concurrently could underline that immunologic, metabolic, or genetic disorders represent the vital cause of these abnormal changes.

## 2. Contemporary Definition and Etiopathogenesis—The State of the Art

This work was performed on a survey of articles collected by exploring the PubMed, GoogleScholar, EMBASE, and MEDLINE databases utilizing the following keywords: “backwash ileitis”, “terminal ileitis”, “ulcerative colitis”, “UC-associated ileitis”, “primary sclerosing cholangitis”, “colonic neoplasia”, “pouchitis”, and “proctocolectomy” in all possible combinations. A retrospective search was carried out to find relevant studies in the field. We extracted information regarding a complete diagnosis (clinical, endoscopic, histopathological, and imaging features) of BWI. We summarized current knowledge related to the topic to promote further research that can improve understanding and help develop clinical practice guidelines for better disease management and control.

The term “backwash” was described in the literature as early as 1936 by Crohn and Rosenak, suggesting that the ileal inflammation observed in severe colitis is due to retrograde colonic contents secondary to the destruction of the ileocecal valve (ICV). The study indicated that these changes were found in some patients with a possible previously undescribed “combined form of ileitis and colitis” [8]. The exact mechanism of this condition was never clearly explained. The hypothetical pathophysiology for this phenomenon suggests that severe inflammation of the proximal cecum/ileocecal mucosa may lead to the malfunction of the ICV that retrogrades colonic material into the distal terminal ileum and, consequently, ileitis [9].

However, despite lacking evidence for backwash as the primary etiopathogenesis for this condition, the term “backwash” ileitis has become notorious in the literature [10,11]. Recent evidence suggests the presence of BWI in up to 35% of patients with UC [12]. Over time, different definitions of BWI have been described depending on the length of ileal involvement (any length from 0.5 cm above the ileocecal valve) [13]. According to recent evidence against BWI theory in UC in favor of primary UC involvement, an updated definition of BWI in the context of a more precise distinction of BWI from CD or other ileitis is needed.

More recently, Patil et al. [12] proposed in their study that the term “UC-associated ileitis” be used instead of BWI, as evidence of primary small bowel/ileal involvement in UC has been increasingly documented. However, studies to date do not provide solid evidence regarding the change in nomenclature of the original terminology. Therefore, further research is needed. Table 1 presents the controversial studies that could change the perspective of the proposed initial “backwash” theory. Initially, we found 10 studies for interpretation. In total, 1/10 had no pathological evaluation to confirm the diagnosis, and in 4/10 studies, patients were likely to have CD and not BWI. Finally, 5/10 studies referred to ileitis associated with UC as the primary UC involvement against the backwash theory of ileitis in UC.

In addition, as many of the UC patients with the initial diagnosis of “BWI” had changed the diagnosis to CD over time [14], the existence of BWI as a distinct disorder has been questioned since its initial description. Also, there is no clear or convincing evidence that ileocecal reflux produces disease in the ileum, either in regular patients or in those with IBD.

**Table 1:** The controversial studies that could change the perspective of the proposed initial “backwash” theory.

Study	Type of Study	No. of Patients	Characteristics Against “BWI”	Study Conclusion
Abdelrazeq [9] (2005)	Prospective	100	- In total, 22 of the patients had ileal involvement - The presence of “BWI” did not correlate with the severity of colitis	- Total proctocolectomy with ileal-pouch anal anastomosis; all patients had pancolitis - Likely primary involvement of UC in the ileum
Haskell [4] (2005)	Retrospective	200	- All patients had a colectomy for medically refractory disease - In total, 34 of the patients had ileal inflammation - In total, 60% of patients with ileal inflammation did not have cecal involvement - In total, 46% of patients showed only mild to moderate activity in the colon	- The activity in the colon was not severe enough to induce incompetence of the ICV
Goldstein [15] (2006)	Retrospective	250 UC 100 CD	- In UC patients, the degree of inflammatory activity in the ileum correlated similarly with that in the colon - In total, 47 cases of UC patients showed quiescent or only mild inflammation of the cecum	- Ileitis in UC likely represents primary UC involvement - CD cohort had distinct features, including more activity in the ileum versus the colon
Yamamoto [16] (2008)	Prospective case control	50	- In patients with BWI and non-BWI, the levels of inflammatory cytokines (IL-1, IL-6, IL-8, and TNF-alfa) were significantly elevated compared with the control group - In total, 8 of 12 cases with ileal involvement had a completely normal cecum and were thought to represent “non-backwash” ileitis	Endoscopic aspect: - In total, 38 patients (76%) had normal TI - In total, 4 patients (8%)—BWI - In total, 8 patients (16%) had non-backwash ileitis (ileitis without cecum involvement) Reflux of colonic contents may not be the only mechanism contributing to the etiology of ileal inflammation in UC
Hamilton [17] (2016)	Prospective case control	72	- In total, 16 of the UC patients had ileitis, but only 6 had inflammation of the cecum, and none showed a dilated or patulous ICV	- Ileitis was not associated with NSAID use, UC-related - Medications, bowel preparation, or infection

BWI: backwash ileitis; UC: ulcerative colitis; CD: Crohn’s disease.

## 3. Clinical Significance and Differential Diagnosis

Routine intubation of the TI is not necessary in mild to moderate UC. However, it should be performed as part of a complete colonoscopy when needed, not only for differential diagnosis but also for assessing disease management, complications, and prognosis in UC [18].

The first clue for diagnosing BWI is the endoscopic appearance of the ICV. The “fish-mouth” presentation of the ICV, associated with BWI, refers to a gaping appearance, likely caused by the loss of the normal ICV tone. A smooth, tubular ICV without stenosis or deformity, allowing easy endoscope passage, contrasts with the narrowed and irregular ICV seen in CD [13].

The macroscopic view of BWI, often observed in moderate-to-severe pancolitis, reveals a short, continuous segment of diffuse erythema, edema, granularity, and friability, with or without superficial erosions and loss of the vascular pattern, resembling the mucosa of the right colon [19]. Unlike CD ileitis, which typically presents with linear ulcerations, cobblestoning, deep ulcers, or strictures, BWI remains a superficial inflammation, confirmed by biopsy specimens [20].

Differentiating BWI from Crohn’s ileitis is crucial, as the two conditions share overlapping clinical and endoscopic features but have distinct histopathological and radiological differences [15].

### 3.1. Endoscopic Findings

BWI typically presents as a continuous inflammation extending from the cecum into the terminal ileum, whereas CD ileitis often manifests with skip lesions and segmental involvement [21]. Endoscopically, BWI is associated with diffuse erythema, granularity, friability, and superficial ulceration without deep fissuring or strictures. Conversely, CD exhibits cobblestoning, aphthous ulcers, deep linear ulcerations, and luminal narrowing [22].

Additional endoscopic features distinguishing BWI from CD include a patulous ileocecal valve (ICV) in BWI, often accompanied by the reflux of colonic contents into the ileum [15]. In contrast, CD is more likely to present with stenosis of the ICV due to fibrosis and chronic inflammation, leading to a narrowed or ulcerated appearance [23]. Histologic confirmation is necessary for cases where endoscopic findings are inconclusive. In BWI, mucosal inflammation remains superficial and continuous, extending from the inflamed cecum, while CD often exhibits patchy involvement with areas of normal mucosa between affected segments [21].

Several other conditions may mimic BWI on endoscopy, including lymphoid hyperplasia, vasculitis, eosinophilic enteritis, NSAID-induced enteropathy, radiation enteritis, and infectious ileitis [24]. Lymphoid hyperplasia is often incidental but can sometimes lead to nodular mucosal changes resembling inflammatory disease. Vasculitic conditions, such as Henoch–Schönlein purpura or Behçet’s disease, may present with ulceration and hemorrhagic necrosis of the ileal mucosa [24].

### 3.2. Histologic Findings

Histological examination plays a critical role in differentiating BWI from CD. BWI is characterized by superficial mucosal inflammation, villous atrophy, and increased lamina propria cellularity without transmural inflammation, granulomas, or neural hyperplasia [15]. In contrast, CD is associated with transmural inflammation, granulomas, pyloric metaplasia, and crypt architectural distortion, findings absent in BWI [22]. Additionally, mucous gland metaplasia and isolated ileal erosions are more characteristic of CD than BWI [15].

More recent histologic studies suggest that pattern recognition of inflammation and crypt architecture can aid in differentiating these conditions [23]. BWI is often linked to pancolitis and right-sided colonic involvement, whereas CD frequently exhibits focal crypt abscesses, fissuring ulcers, and transmural inflammation [21]. Moreover, cases of chronic BWI may exhibit progressive crypt atrophy and mucosal distortion, which may overlap with early-stage CD [22].

Lymphoid hyperplasia, though often benign, can histologically resemble CD, necessitating careful evaluation of crypt distortion and granulomas to exclude IBD [24]. Additionally, vasculitis-related ileitis may show leukocytoclastic vasculitis and fibrinoid necrosis, which are absent in CD [25].

The histological pattern may suggest acute or chronic ileitis in both BWI and CD ileitis. In BWI, the inflammation is limited to the mucosa, with villous atrophy and scattered crypt abscesses. Still, transmural lymphoid aggregates, noncaseating granulomas, and fissuring ulcers indicate CD ileitis [15,22].

### 3.3. Imagistic Findings

Backwash ileitis (BWI) can be visualized on cross-sectional imaging, mainly in patients with pancolitis, as segmental inflammation of the terminal ileum. This aspect can complicate the diagnostic process and raise concerns about proper management. As most cases appear in the context of moderately to severely active UC, the imaging features that should raise suspicion about this condition include extensive inflammation of the entire colon with involvement of the terminal ileum [26,27,28].

Current practice in the field of IBD focuses on using non-invasive explorations for a complete evaluation of disease extension and follow-up of response to treatment. Abnormal parameters involving the bowel wall thickness (BWT), stratification (BWS), and vascularity can also be extrapolated for this condition. Although non-specific, the continuous colon inflammation, with no skip lesions, suggests UC; thus, terminal ileum involvement requires differentiation between BWI and terminal ileum involvement from CD. In some rare cases, ileitis was observed in patients with only left-sided colonic involvement, thus possibly delaying the correct diagnosis and proper treatment [27,29].

Historically, barium studies used to evaluate the digestive tract included small bowel follow-through and barium enema. The features of BWI, such as a widely patent ileocecal valve and persistent dilatation of the terminal ileum, lead to the hypothesis that the inflammation was caused by the reflux of colic content due to a pathologically enlarged ileocecal valve [27]. These studies have been replaced in the modern era by computed tomography (CT) and magnetic resonance enterography (MRE) due to their capability to assess the bowel wall and surrounding tissues.

Intestinal ultrasound (IUS) is an easily accessible, non-expensive, and non-radiating tool that can detect an increased BWT, color Doppler signal (as a sign of increased vascularity), and loss of the BWS. However, its accuracy for evaluating the terminal ileum depends on a series of factors, such as the patient’s preparation and the experience of the clinician or radiologist. In addition, due to the low specificity of these parameters, more studies have focused on using MRI for a more appropriate disease characterization.

CT exploration of the bowel wall is highly dependent on bowel distension. It is usually performed only in acute clinical settings because of the radiation-associated risks, but it can offer valuable information, similar to MRE. Pathological findings are identical to those of IUS; the lead pipe appearance of the colon is highly suggestive of UC, which, in association with the clinical aspects and the presence of terminal ileum inflammation, should raise suspicion of BWI [30].

MRI is one of the best imaging methods available for evaluating patients with IBD, both in the initial phase to assess the extent of inflammation in the small bowel and for following up with the patients under treatment [31]. Only a few studies on the use of MRE for BWI have been published to date, suggesting the need for extensive research for this condition. Patient preparation is thorough and essential for obtaining diagnostic images, requiring complete patient cooperation. The proper bowel distention can be tested by obtaining a T2 sequence and supplementing the intraluminal fluid with additional contrast [32]. The specific differentiation of terminal ileum inflammation between UC and CD can only be made with a combination of ileocolonoscopy and clinical parameters. However, MRE can offer a series of parameters that are more suggestive of BWI than the terminal ileum involvement of CD. For instance, the BWT of the TI in patients with CD is significantly higher than in those with BWI, and the difference in the transmural nature of CD can explain it. In addition, eccentric thickening was observed in patients with CD compared to the circumferential involvement of BWI. The length of the diseased terminal ileum was also significantly longer in patients with CD. As mentioned before, the patency of the ICV can be evaluated by imaging: the gaping of this valve, measured as the distance between its lips, was significantly higher in those with BWI than those with terminal ileitis due to CD. The patency index, which is more accurate due to normalizing the ileocecal valve gaping to the intraluminal diameters of the terminal ileum or cecum, is also statistically significant when used to characterize BWI. Symmetrical enhancement of the inner wall of the terminal ileum is another feature of BWI that can differentiate it from CD. At the same time, no difference in the diffusion restriction at this level was observed between the two. Terminal ileum distension was observed more frequently in BWI, while the frequency of “comb sign” and lymph node involvement was significantly higher in those with CD [27,32].

### 3.4. Additional Diagnostic Modalities

Fluoroscopic small bowel follow-through and capsule endoscopy can further assist in differentiating these conditions. In BWI, imaging findings are localized to the terminal ileum, with no evidence of deep ulceration or fibrosis [15]. CD, however, frequently involves multiple segments of the small intestine, with characteristic cobblestone mucosa, strictures, and deep ulcerations [21]. MRE studies suggest that while BWI may be identified by ICV gaping and terminal ileum dilation, these features alone are unreliable differentiators from other causes of ileitis [27].

Backwash ileitis must be distinguished from CD, infectious ileitis, ischemic ileitis, NSAID-induced enteropathy, radiation enteritis, neoplastic conditions, lymphoid hyperplasia, and vasculitis. Histopathological findings, radiologic imaging, and endoscopic assessment provide key diagnostic clues, and a multimodal approach is essential for accurate differentiation. In cases where histopathology is inconclusive, molecular and microbiological testing should be considered to rule out infectious and neoplastic mimics [15].

A positive diagnosis of BWI in patients who are scheduled for proctocolectomy is considered a risk factor for developing postoperative Crohn’s disease-like pouch complications. Although pre-operative CT or MRE does not influence the surgical results and the rate of complications [29], they can demonstrate the extent of the disease and other manifestations or complications, which is essential for a better understanding of the patient’s condition.

## 4. The Relationship of BWI with CRC, PSC, and Pouchitis

**Figure 1:**
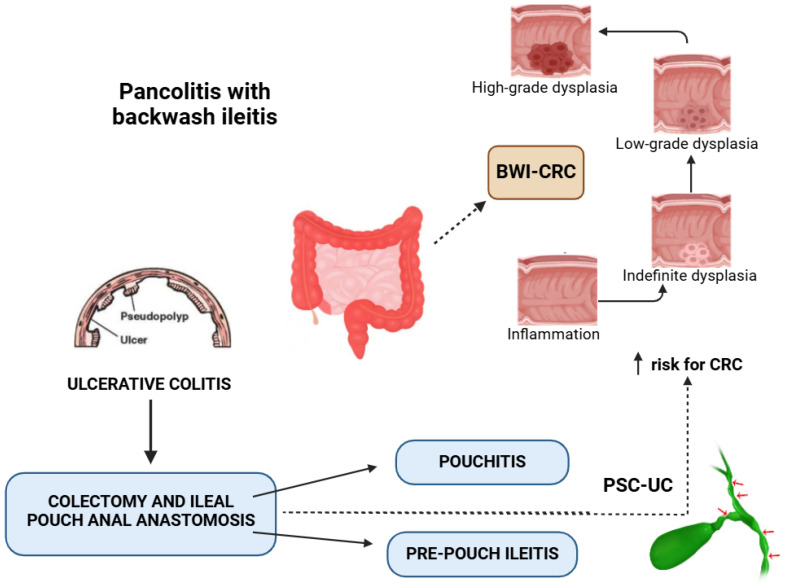
BWI-CRC: backwash ileitis–colorectal cancer; PSC-UC: primary sclerosing cholangitis–ulcerative colitis. ↑ risk for CRC = increased risk for colorectal cancer; ↑: multiple strictures of the biliary tree.

Backwash ileitis in UC patients may represent a potential risk factor for neoplasia, a positive association with primary sclerosing cholangitis, and a higher risk for the subsequent development of pouchitis after restorative proctocolectomy; Figure 1 illustrates the link between these entities.

### 4.1. BWI and the Risk of CRC

The relationship between ileal inflammation in patients with UC and the risk of CRC development has been assessed in several studies in the literature. Still, the results of these studies are contradictory [4,33,34,35]. When first described, ileitis in UC patients was believed to be the consequence of extensive and severe disease that affected the ileocecal junction and led to the retrograde flow of colonic contents [36,37]. Considering this hypothesis, the association of BWI with CRC can be explained through the continuous cycle of inflammation, healing, and re-inflammation that can lead to dysplasia and cancer. It is well known that the long duration and extent of disease are the risk factors for malignant transformation in UC patients [38].

Research on the association of UC and BWI suggests that these increase the risk for CRC. One of these studies, by Heuschen et al. [33], reported a strong association between BWI and CRC that undergo proctocolectomy. This was a prospective study involving 590 consecutive UC patients with restorative proctocolectomy. CRC was found in 11.2% of all 590 patients. The frequency of CRC was significantly higher (29.0%) in the group of patients with pancolitis and BWI compared with patients with pancolitis without BWI (9.0%) and patients with left-sided colitis (1.8%). Additionally, CRC patients with pancolitis and BWI had significantly more multiple tumor growth (45.2%) than patients with pancolitis without BWI (24.2%) and left-sided colitis (0%). The data presented by Heuschen et al. were afterward questioned, considering that the number of patients previously diagnosed with CRC in the three study groups was not equal.

Another known risk factor for CRC in patients with UC is the presence of primary sclerosing cholangitis (PSC). In the study of Heuschen et al. [33], PSC was strongly associated with BWI, yet interestingly, it was not related to the development of CRC. However, in other studies, PSC has been considered a potential risk factor for malignancy transformation in patients with UC [39].

Alterations in the enterohepatic circulation of cholic acids are theorized to be implicated in the tumorigenesis of CRC [40]. The terminal ileum is the site of cholic acid absorption, which may indicate their potential involvement in the development of BWI in patients with UC. Likewise, the mucosal inflammation from BWI could also alter the enterohepatic circulation of cholic acids.

Another study that sustained the association between BWI and the risk of colonic neoplasia was conducted by Navaneethan et al. [34]. It was a prospective study that included 4198 UC patients who underwent colectomy. The results of this research revealed that endoscopic and histologic features (the grade of dysplasia) that predict colonic neoplasia were more frequent in BWI patients, suggesting that BWI may be a risk factor for CRC. Moreover, in the study mentioned above [34], PSC was found to be an independent risk factor for colonic neoplasia in UC patients with proctocolectomy. Despite this, BWI was not related to the malignant development of CCR when colon cancer alone was investigated (5.6% of patients with colon cancer in the BWI group compared to 3% in the control group, *p* = 0.14). In this study, there was also no difference in the frequency of multiple tumors between the BWI group and the control groups.

Other research from the literature failed to establish an actual connection between BWI and CRC. The study of Haskell et al. [4] showed a similar low rate of dysplasia and carcinoma in patients with BWI compared with the controls, suggesting that patients with BWI do not have an increased risk of developing colorectal neoplasia.

Furthermore, Rutter et al. [35] conducted a case-control study involving 204 UC patients in which cancer surveillance was evaluated. The results of his research showed no significant association between BWI and cancer risk in UC cases after 5 years of follow-up. BWI was found mainly in patients with chronic extensive disease that involved the ICV. Even though the odds ratio of neoplasia was 2.5 for patients with BWI, this result was not appreciated as significant because not all patients had terminal ileal intubation. Table 2 resumes the studies about BWI and CRC risk.

### 4.2. BWI and PSC

BWI and PSC are considered to be two distinct conditions often associated with IBD, particularly with UC. The exact link between these conditions is not entirely understood. Although PSC-IBD shares specific common characteristics that could argue for the existence of a single condition, taken separately, none represent a solid argument.

PSC has an incidence of 0.07–1.3/100,000 people, and IBD is diagnosed in 70% of these cases (from which 75% are with UC). PSC is present in 2-14% of patients with IBD. UC associated with PSC has been considered to have a distinctive phenotype characterized by pancolitis, BWI, rectal sparing, and increased risk for CRC.

While several studies have shown an increased prevalence of BWI in patients with UC and PSC [41,42,43,44], others have not [45,46] (Table 3).

**Table 3:** The prevalence of BWI in PSC-UC patients.

Studies	PSC-IBD (No. of Cases)	BWI (%)
Loftus et al. [41] (2005), United States, Gut.	71	51
Sokol et al. [47] (2008), United States, World J Gastroenterol.	75	18.7
Joo et al. [42] (2009), Korea, Am J Surg Pathol.	40	35.7
Ye et al. [43] (2011) Korea, Inflamm Bowel Dis.	21	42.9
Jørgensen et al. [47] (2012), Norway, Inflamm Bowel Dis.	155	20
Boonstra et al. [45] (2012), Netherlands, Inflamm Bowel Dis.	80	5
Sinakos et al. [46] (2013), United States, Inflamm Bowel Dis.	129	11.6
Navaneethan et al. [34] (2013), United States, Dig Dis Sci.	31	17.4
Murasugi S et al. [44] (2020), Japan, Gastroenterol Res Pract.	35	37

UC: ulcerative colitis; BWI: backwash ileitis; PSC: primary sclerosing cholangitis.

Clinical features in PSC-IBD were first evaluated in 2005 in a paper by Loftus et al., who described a unique phenotype of IBD in these patients, characterized by a high frequency of pancolitis, rectal sparing, and BWI. In this study, BWI was more frequent in the PSC-IBD group (51%) than in the control group (7%) [41].

Subsequent studies were performed describing the distinctive phenotype of IBD with PSC. In 2008, Sokol et al. [48] confirmed that patients with PSC-IBD have a particular disease phenotype, BWI, which is found in 18.7% of patients. Although these patients had a milder form of the disease, they presented a higher risk of CRC.

PSC has been considered a potential risk factor for CRC in UC patients. The pathophysiological mechanism responsible for PSC alters the enterohepatic circulation of cholic acids. The same mechanism is considered to be a risk factor for the occurrence of CRC. Taking into account that the absorption of cholic acids takes place in the terminal ileum, they can be considered to be involved in the occurrence of BWI in patients with UC. On the other hand, ileal inflammation in BWI can also lead to alterations in the enterohepatic circulation of cholic acids [49].

In two studies from Korea (2009, 2011), BWI was more frequently described in PSC-UC patients (35.7% and 42.9%), and the risk of developing CRC was also higher in these patients [42,43]. Heuschen et al. [33] reported that BWI was frequent in PSC-UC patients (26.7%), but it was not associated with the development of CRC in these patients. Jørgensen et al. [47] reported that BWI was seen in 20% of PSC-UC patients. His study showed a significant difference between BWI’s endoscopic and histologic findings. Ileal inflammation was found in 12% of cases in endoscopy and 20% of cases in histology. The right colon was also involved in patients with confirmed histologic ileal inflammation. The colonic inflammatory changes were considerably higher in these patients than those without BWI [47].

Disparities between endoscopic disease activity and clinical manifestations were described to be more frequent in patients with PSC-UC compared to UC alone. Some authors consider that the presence of BWI may be overestimated due to the frequent involvement of the right colon in PSC-UC compared to UC controls. As stated by the IPSCSG (International PSC Study Group) consensus panel on definitions, there are insufficient data to define BWI as a typical PSC-IBD phenomenon [50]. Another study reported a low prevalence of BWI in PSC-UC patients (6%), with no statistical differences between the control group (2%—UC without PSC) [45]. A more recent Korean study by Murasugi et al. reported more moderate/severe UC associated with PSC than previous studies. BWI was found in 37% of PSC-UC cases. Similar to earlier studies with the PSC-UC cases, patients presented pancolitis, occasionally with BWI and rectal sparing [44].

### 4.3. BWI and Ileoanal Pouch-Related Complications

The impact of BWI on the development of pouchitis after IPAA was first assessed in the study of Gustavsson et al. in 1987 [51]. This research included 131 patients with proctocolectomy and IPAA. Twenty patients were diagnosed with pouchitis after the intervention, and only 10% presented previous BWI. According to the results of this study, previous BWI did not influence the development of pouchitis.

The literature data regarding the relationship between BWI and pouchitis in patients with IPAA remain vague. Some studies reported higher rates of pouchitis in patients with BWI [29,52], while others have shown that BWI does not affect the outcome of patients with IPAA [53,54,55].

The study of Urquhart et al. investigated the incidence of pouch neoplasia in IBD patients following IPAA. From a total of 1319 patients, 95.2% had UC, and BWI was found in 14.4% of patients before IPAA. The presence of BWI, extensive colitis, and PSC before IPAA was identified as potential factors for the risk of pouch neoplasia [52].

Recent research by Freund et al. [29] reported that BWI is a predictive factor for later developing Crohn’s disease-like pouch complications (CDLPCs).

Furthermore, Jillawar et al. reported that BWI was not among the risk factors that were statistically associated with complications after restorative proctocolectomy with IPAA [53]. White et al. prospectively compared the outcome of IPAA in colitis patients with BWI and without BWI. The reported results showed that the incidence of acute/chronic pouchitis and de novo Crohn’s disease after IPAA did not differ significantly between patients with or without BWI [54]. Arrossi et al. [55] reported that BWI does not affect pouch outcomes in patients undergoing restorative proctocolectomy.

## 5. Current Therapy and Future Directions

Managing BWI requires medical therapies, lifestyle modifications, close monitoring, and, in some cases, surgical intervention. Given the fact that BWI appears in the context of UC, the treatment is similar but may require a more aggressive approach, depending on the severity of the disease and ileal involvement. There are no specific treatments for BWI. Regardless of the extension of the lesions in the colon associated with BWI, the treatment is similar to that of pancolitis. As described and mentioned above, therapy also focuses on related complications and associated diseases.

Severe UC with marked ileal inflammation describing BWI underscores the complexity and severity that UC can present when inflammation extends into the TI. In this condition, management of the disease focuses on controlled therapy of the extensive mucosal inflammation and close monitoring (laboratory tests, imaging, and endoscopic evaluations) to assess treatment efficacy. Accurate recognition and diagnosis of severe UC with BWI is critical for preventing complications and optimizing treatment.

In the literature are also mentioned two cases of BWI complicated by massive hemorrhage and perforation that required surgical intervention. In both cases, staged operation without sacrificing the involved area of the terminal ileum led to ileal pouch–anal anastomosis, with a favorable postoperative outcome [56].

Although laboratory tests alone cannot confirm BWI, they provide supportive evidence. Common inflammatory markers of active inflammation, such as elevated C-reactive protein and erythrocyte sedimentation rate, are not disease-specific [57]. Fecal Calprotectin appears to be the most popular and well-studied non-invasive biomarker in diagnosing and monitoring IBD patients [58]. Also, a Complete Blood Count may reveal anemia or leukocytosis in active disease. At present, no biomarker is accurate enough to replace endoscopy with biopsies. Although more assessments should focus on specific biomarkers, comparative studies and more clinical trials are also needed to establish the full potential of new biomarkers to impact clinical care.

Findings in the literature have changed the traditional dogma that UC affects only the colon, and inflammatory changes in the ileum have likely been described as primary UC involvement instead of the “backwash” theory (changes due to ICV incompetence). The term “UC-associated ileitis” proposed by Patil et al. [12] to be used instead of “BWI” needs validation; therefore, future research that contradicts the “backwash” theory and its clinical implications should be conducted using strict and uniform criteria.

The emphasis on TI intubation and colonoscopic surveillance should also be considered. Routine intubation of the TI should be performed as part of a complete colonoscopy, not only for differential diagnosis but also for assessing disease management, complications, and prognosis in UC.

## 6. Conclusions

In conclusion, the traditional dogma that the only mechanism causing BWI is the severe inflammation leading to ICV incompetence has been changed. Published evidence in the last decades considered BWI as a primary manifestation of UC involving the ileum. The controversy over whether BWI is a misnomer or considered ileitis likely with primary UC involvement needs to be clarified by further research in this field. Associated ileitis in UC patients predisposes them to a more refractory course of the disease; therefore, establishing a correct and complete diagnosis of the disease leads to better management of the disease, avoiding associated complications.

## Figures and Tables

**Table 2:** Studies about UC-BWI and CRC.

Study	No. of UC Cases	BWI	CRC/Dysplasia in UC-BWI Patients	Study Conclusion
Heuschen et al. [33] (2001)	590	18.13%	29%	- Strong association between BWI and CRC in UC
Rutter et al. [35] (2004)	204	12% - Terminal ileal intubation had been achieved only in 70% of patients, of whom 12% had BWI	4.8% (result was not significant)	- Association between BWI and the risk of colonic neoplasia in the colectomy specimen - BWI was not related to the malignant development of CCR when colon cancer alone was investigated
Haskell et al. [4] (2005)	200	17%	0%	- BWI in UC is not associated with an increased risk of cancer at UC
NavaNathan et al. [34] (2013)	4198	24.9%	18%	- BWI with extensive colitis was associated with the risk of identifying colon neoplasia but not cancer alone in the proctocolectomy specimen.

BWI: backwash ileitis; UC: ulcerative colitis; CRC: colorectal cancer.
